# Supplementary material for: Trans-basin Atlantic-Pacific connections further weakened by common model Pacific mean SST biases
Source: Nat Commun. 2020 Nov 10;11:5677. doi: 10.1038/s41467-020-19338-z (PMC7655828; doi:10.1038/s41467-020-19338-z)
Supplement: Supplementary file 1 — Supplementary Information [file 41467_2020_19338_MOESM1_ESM.pdf]

Supplementary information for

Trans-basin Atlantic-Pacific connections further weakened by common model Pacific mean SST biases

Chen Li*, Dietmar Dommenget, Shayne McGregor

ARC Centre of Excellence for Climate Extremes, School of Earth Atmosphere and Environment, Monash University, Clayton 3800, Victoria, Australia

Supplementary Table 1: Experiments design for the partially coupled (PARCP) UM7.3 (N48L38) model. The atmosphere model (UM7.3) is coupled with a slab mixed-layer ocean in the Pacific basin, while the Atlantic and Indian Ocean are prescribed with 12-monthly varying sea surface temperature (SST). Note the Indian Ocean has been prescribed to the observed climatology for all experiments. The first 10 years model outputs are excluded for the following analysis.

PARCP	Pacific (slab ocean)	Atlantic (prescribed)	Run length
EXP1_Obs.	Obs. Clim.	Control: Obs. Clim.<br>Warm: Obs. Clim. + warm pattern	100 years<br>100 years
EXP2_Atlantic bias	Obs. Clim.	Control: CMIP5 Clim.<br>Warm: CMIP5 Clim. + warm pattern	100 years<br>100 years
EXP3_Pacific_bias	CMIP5 Clim.	Control: Obs. Clim.<br>Warm: Obs. Clim. + warm pattern	100 years<br>100 years
EXP4_AtlPac_bias	CMIP5 Clim.	Control: CMIP5 Clim.<br>Warm: CMIP5 Clim. + warm pattern	100 years<br>100 years

Supplementary Table 2: Regional averaged sea surface temperature (SST) and Pacific trade wind responses to the Atlantic warming forcing in different background experiments, including the SST response (Unit: °C) in the equatorial and south off-equatorial Niño3.4 region, and the zonal wind stress response (Unit: N m^{-2}) in the equatorial central Pacific. Negative zonal wind stress represents the anomalous easterly response, thus indicates the strengthening of Pacific trade wind.

PARCP	Niño3.4 (5S-5N, 170W-120W)	South off-equatorial Niño3.4 (10S-Eq., 170W-120W)	Central Pacific zonal wind stress (6S-6N,160E-140W)
EXP1_Obs.	-0.28	-0.22	-4.7×10^{-3}
EXP2_Atlantic bias	-0.18	-0.12	-2.7×10^{-3}
EXP3_Pacific_bias	-0.19	-0.08	-2.2×10^{-3}
EXP4_AtlPac_bias	-0.03	0.03	-2.5×10^{-3}

Supplementary Table 3: Experiments design for the atmosphere-only model (AGCM). Same as Supplementary Table 1, but turning off the slab ocean in Pacific.

AGCM	Pacific (prescribed)	Atlantic (prescribed)	Run length
EXP1_Obs.	Obs. Clim.	Control: Obs. Clim.<br>Warm: Obs. Clim. + warm pattern	100 years<br>100 years
EXP2_Atlantic bias	Obs. Clim.	Control: CMIP5 Clim.<br>Warm: CMIP5 Clim. + warm pattern	100 years<br>100 years
EXP3_Pacific_bias	CMIP5 Clim.	Control: Obs. Clim.<br>Warm: Obs. Clim. + warm pattern	100 years<br>100 years
EXP4_AtlPac_bias	CMIP5 Clim.	Control: CMIP5 Clim.<br>Warm: CMIP5 Clim. + warm pattern	100 years<br>100 years

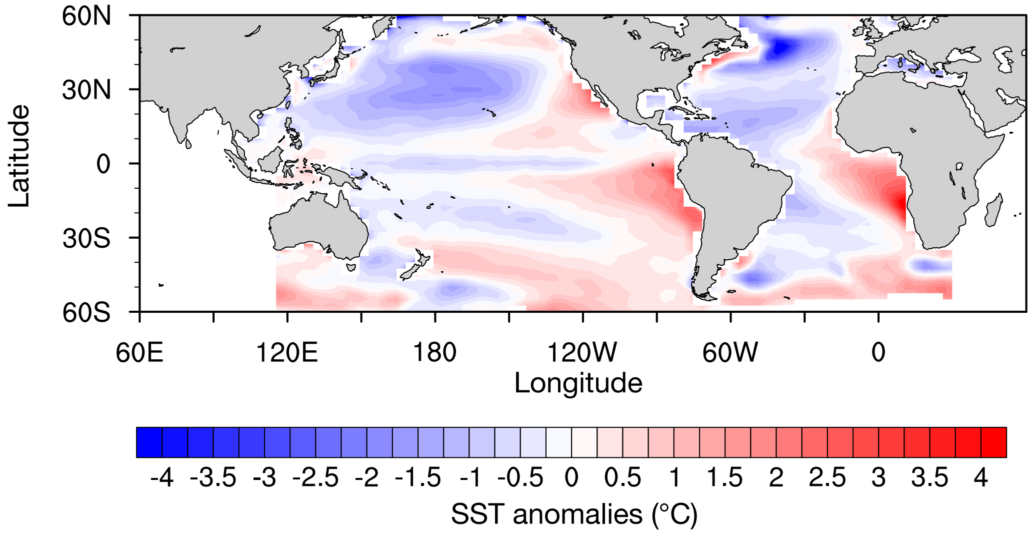

Supplementary Figure 1: Climatological (1980-2005) annual mean sea surface temperature (SST) difference (unit: °C) between the multi-model ensemble mean of 46 CMIP5 (Coupled Model Intercomparison Project phase 5) models and the observed SST (HadISST1) for the Pacific and Atlantic region.

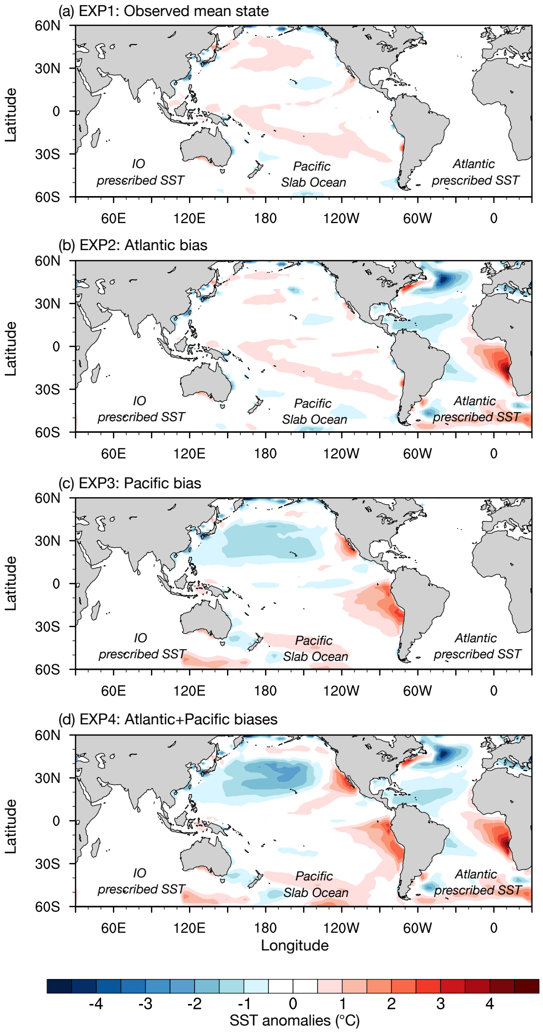

Supplementary Figure 2: Annual mean sea surface temperature (SST) difference (unit: °C) between the partially coupled (PARCP) control runs and HadISST of, **a** observed mean state (unbiased) simulation (experiment 1, EXP1), **b** Atlantic bias simulation (experiment 2, EXP2), **c** Pacific bias simulation (experiment 3, EXP3), and **d** combined Atlantic and Pacific biases simulation (experiment 4, EXP4).

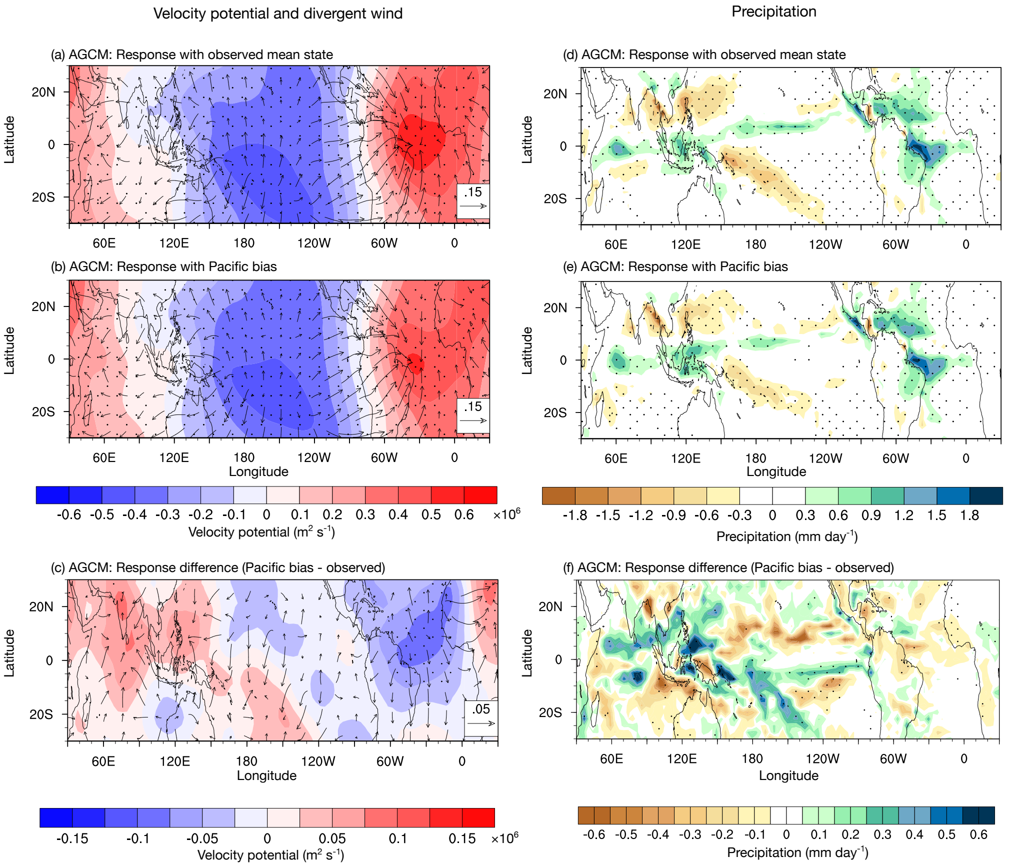

Supplementary Figure 3: a, b Low-layers (950-500hPa) averaged velocity potential (shading; m^2 s^{-1} scaled by 10^6) and divergent wind (vectors; m s^{-1}) responses to Atlantic warming forcing in unbiased and Pacific bias atmosphere-only (AGCM) simulations, respectively. **c**, the response difference between the Pacific bias (**b**) and the unbiased (**a**) simulation. **d-f**, as (**a-c**) but for the precipitation response (unit: mm day^{-1}). Stippling indicates the statistical significance at the 10% level.

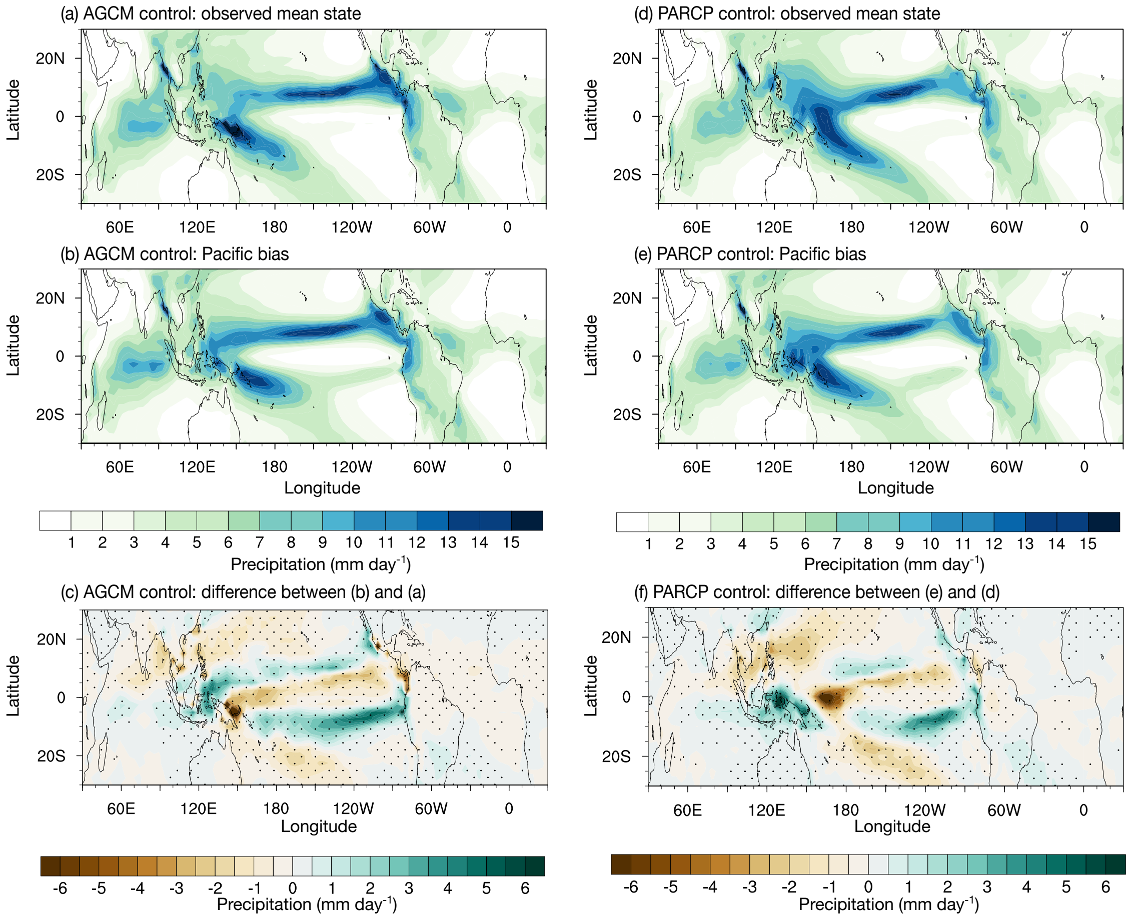

Supplementary Figure 4: a, b The climatological annual mean precipitation (mm day^{-1}) in unbiased and Pacific bias atmosphere-only (AGCM) control runs, respectively. **c**, the difference between the (**b**) and (**a**). **d-f,** as in (**a-c**), but for the partially coupled (PARCP) control runs. Stippling in (**c**) and (**f**) indicates the difference significance at the 10% level. The double ITCZ problem under the CMIP5-like Pacific SST background can be seen from both atmosphere-only and slab-ocean coupled simulations.

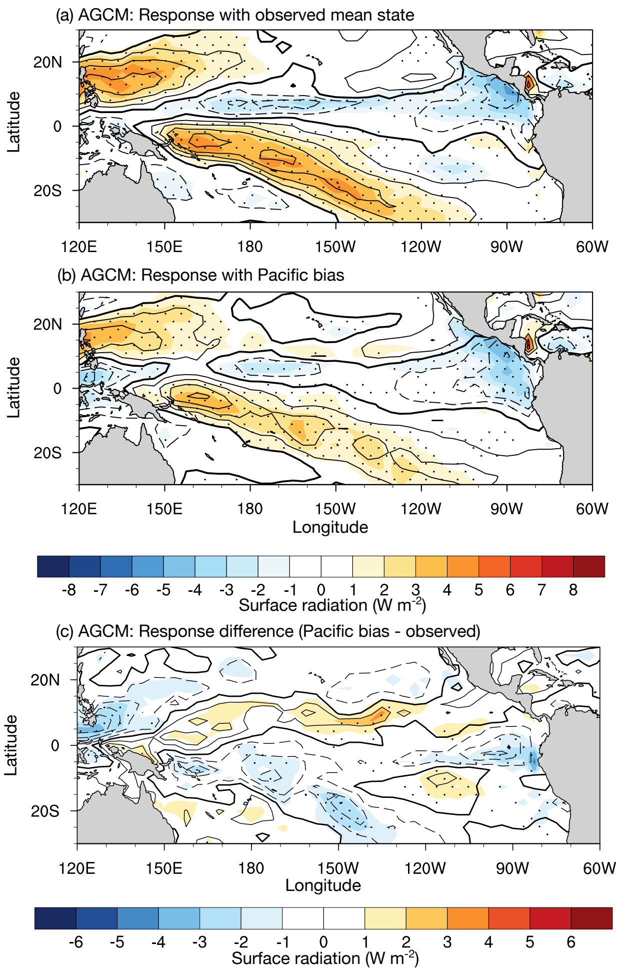

Supplementary Figure 5: a, b The surface downward shortwave and longwave radiation (shading; W m^{-2}) and outgoing longwave radiation (OLR) (contour; W m^{-2}) responses to Atlantic warming forcing in unbiased and Pacific bias atmosphere-only (AGCM) simulations, respectively. **c**, the response difference between the (**b**) and (**a**). The solid (dashed) contour indicates positive (negative) value for OLR, which refers to less (more) clouds. Solid bold contour indicates 0. Contour interval for OLR is 1.5 and 1 W m^{-2} in (**a, b**) and (**c**), respectively. Stippling indicates the statistical significance at the 10% level for surface radiation.

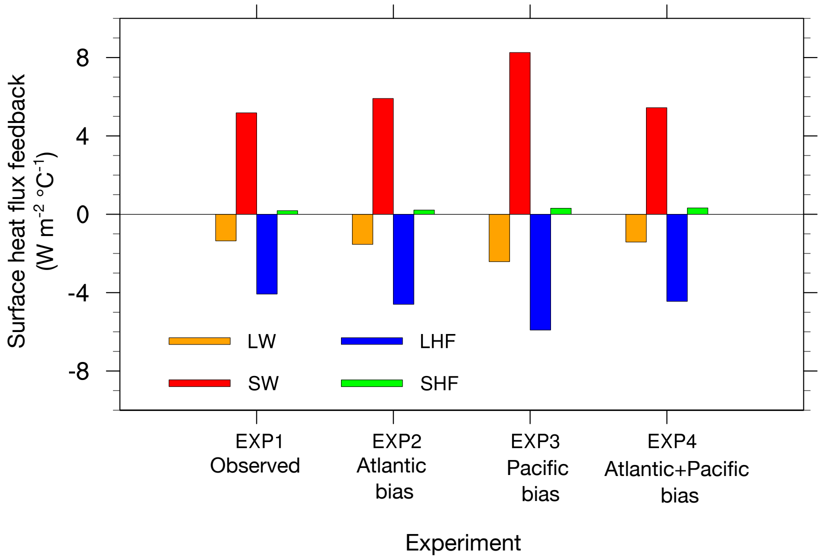

Supplementary Figure 6: Individual surface heat flux feedback components, which represented by the regression between the sea surface temperature (SST) and surface heat flux, over the eastern Pacific (Niño3 region: 5°S-5°N, 150°-90°**W**) in the partially coupled (PARCP) control runs.
