# Supplementary material for: Trans-basin Atlantic-Pacific connections further weakened by common model Pacific mean SST biases
Source: Nat Commun. 2020 Nov 10;11:5677. doi: 10.1038/s41467-020-19338-z (PMC7655828; doi:10.1038/s41467-020-19338-z)
Supplement: Supplementary file 3 — Reporting Summary [file 41467_2020_19338_MOESM3_ESM.pdf]

nature research

Corresponding author(s): Chen Li

Last updated by author(s): Oct 1, 2020

Reporting Summary

Nature Research wishes to improve the reproducibility of the work that we publish. This form provides structure for consistency and transparency in reporting. For further information on Nature Research policies, see our Editorial Policies and the Editorial Policy Checklist.

Statistics

For all statistical analyses, confirm that the following items are present in the figure legend, table legend, main text, or Methods section.

n/a	Confirmed	
☐	☒	The exact sample size (n) for each experimental group/condition, given as a discrete number and unit of measurement
☐	☒	A statement on whether measurements were taken from distinct samples or whether the same sample was measured repeatedly
☐	☒	The statistical test(s) used AND whether they are one- or two-sided<br>*Only common tests should be described solely by name; describe more complex techniques in the Methods section.*
☐	☒	A description of all covariates tested
☐	☒	A description of any assumptions or corrections, such as tests of normality and adjustment for multiple comparisons
☐	☒	A full description of the statistical parameters including central tendency (e.g. means) or other basic estimates (e.g. regression coefficient) AND variation (e.g. standard deviation) or associated estimates of uncertainty (e.g. confidence intervals)
☐	☒	For null hypothesis testing, the test statistic (e.g. F, t, r) with confidence intervals, effect sizes, degrees of freedom and P value noted<br>*Give P values as exact values whenever suitable.*
☐	☐	For Bayesian analysis, information on the choice of priors and Markov chain Monte Carlo settings
☐	☐	For hierarchical and complex designs, identification of the appropriate level for tests and full reporting of outcomes
☐	☒	Estimates of effect sizes (e.g. Cohen's d, Pearson's r), indicating how they were calculated

Our web collection on statistics for biologists contains articles on many of the points above.

Software and code

Policy information about availability of computer code

Data collection	Numerical model simulation data are used in this study. The atmosphere model, Met Office Unified Model (UM7.3), is coupled with a 50m depth mixed-layer slab ocean in the Pacific basin.
Data analysis	We first used python code to convert model output into netcdf format. Then we used NCAR Command Language (NCL) to generate the figures.

For manuscripts utilizing custom algorithms or software that are central to the research but not yet described in published literature, software must be made available to editors and reviewers. We strongly encourage code deposition in a community repository (e.g. GitHub). See the Nature Research guidelines for submitting code & software for further information.

Data

Policy information about availability of data

All manuscripts must include a data availability statement. This statement should provide the following information, where applicable:

- Accession codes, unique identifiers, or web links for publicly available datasets
- A list of figures that have associated raw data
- A description of any restrictions on data availability

Model outputs that support the findings of this study have been deposited in [http://doi.org/10.5281/zenodo.4054673]
The codes used to analysis the model output and generate all the figures (includes the figures in the supplementary information) are available at [https://doi.org/10.5281/zenodo.4054644]

Field-specific reporting

Please select the one below that is the best fit for your research. If you are not sure, read the appropriate sections before making your selection.

☐ Life sciences ☐ Behavioural & social sciences ☒ Ecological, evolutionary & environmental sciences

For a reference copy of the document with all sections, see nature.com/documents/nr-reporting-summary-flat.pdf

Ecological, evolutionary & environmental sciences study design

All studies must disclose on these points even when the disclosure is negative.

Study description	This study is based numerical model simulation, including two different type of climate models, one is the globally atmosphere-only model and another one is globally atmosphere model - coupled with a mixed-layer slab ocean on the Pacific basin.
Research sample	In total, we did 16 individual simulations and each simulations run for 100 years.
Sampling strategy	The first 10 years model outputs of the total 100 years run are excluded for the statistical analysis, thus, 90 sample sizes (represented by 90 model years) for each simulation are presumed for the significant test.
Data collection	Chen Li did the all the model simulations and analysis the model outputs.
Timing and spatial scale	The data we used for the study is from the model year 10 to 100, globally output with a horizontal grid spacing of 3.75° longitude by 2.5° latitude
Data exclusions	The first 10 years model outputs are excluded for the statistical analysis, as considering as the model spin-up time.
Reproducibility	The experimental design is described in the manuscript test and model code can be access with registration and permission.
Randomization	We considered each model simulation output as one group, the 90 years' model output present for 90 sample member, and the 90-members ensemble mean is used to present the model's mean state, the 90-years standard deviation is used to estimate the significant test.
Blinding	We use all the model output expect the first 10 years (it's common for the climate model study as the model requires some spin-up time to reach the equilibrium state), we didn't select our sample randomly.

Did the study involve field work? ☐ Yes ☒ No

Reporting for specific materials, systems and methods

We require information from authors about some types of materials, experimental systems and methods used in many studies. Here, indicate whether each material, system or method listed is relevant to your study. If you are not sure if a list item applies to your research, read the appropriate section before selecting a response.

Materials & experimental systems

n/a	Involved in the study	
☒	☐	Antibodies
☒	☐	Eukaryotic cell lines
☒	☐	Palaeontology and archaeology
☒	☐	Animals and other organisms
☒	☐	Human research participants
☒	☐	Clinical data
☒	☐	Dual use research of concern

Methods

n/a	Involved in the study	
☒	☐	ChIP-seq
☒	☐	Flow cytometry
☒	☐	MRI-based neuroimaging
